# Adverse Drug Events after Kidney Transplantation

**DOI:** 10.3390/jpm13121706

**Published:** 2023-12-14

**Authors:** Lionel Rostaing, Thomas Jouve, Florian Terrec, Paolo Malvezzi, Johan Noble

**Affiliations:** 1 Nephrology, Hemodialysis, Apheresis and Kidney Transplantation Department, University Hospital Grenoble, 38043 Grenoble, France; tjouve@chu-grenoble.fr (T.J.); fterrec@chu-grenoble.fr (F.T.); pmalvezzi@chu-grenoble.fr (P.M.); jnoble@chu-grenoble.fr (J.N.); 2 Institute for Advanced Biosciences (IAB), INSERM U 1209, CNRS UMR 5309, Université Grenoble Alpes, 38043 Grenoble, France; 3 Ramathibodi Hospital, Mahidol University, Bangkok 10400, Thailand

**Keywords:** kidney transplantation, immunosuppression, adverse drug event, nephrotoxicity, NODAT, anemia, leukopenia, viral reactivation, hypertension, dyslipidemia

## Abstract

*Introduction:* Kidney transplantation stands out as the optimal treatment for patients with end-stage kidney disease, provided they meet specific criteria for a secure outcome. With the exception of identical twin donor–recipient pairs, lifelong immunosuppression becomes imperative. Unfortunately, immunosuppressant drugs, particularly calcineurin inhibitors like tacrolimus, bring about adverse effects, including nephrotoxicity, diabetes mellitus, hypertension, infections, malignancy, leukopenia, anemia, thrombocytopenia, mouth ulcers, dyslipidemia, and wound complications. Since achieving tolerance is not feasible, patients are compelled to adhere to lifelong immunosuppressive therapies, often involving calcineurin inhibitors, alongside mycophenolic acid or mTOR inhibitors, with or without steroids. *Area covered*: Notably, these drugs, especially calcineurin inhibitors, possess narrow therapeutic windows, resulting in numerous drug-related side effects. This review focuses on the prevalent immunosuppressive drug-related side effects encountered in kidney transplant recipients, namely nephrotoxicity, post-transplant diabetes mellitus, leukopenia, anemia, dyslipidemia, mouth ulcers, hypertension, and viral reactivations (cytomegalovirus and BK virus). Additionally, other post-kidney-transplantation drugs such as valganciclovir may also contribute to adverse events such as leukopenia. For each side effect, we propose preventive measures and outline appropriate treatment strategies.

## 1. Introduction

Currently, worldwide, there is a significant burden of end-stage kidney disease (ESKD), with major causes linked to diabetes and hypertension [1,2]. However, there are geographical variations globally, and its incidence and prevalence are increasing worldwide. ESKD represents the ultimate stage of chronic kidney disease (CKD). In 2017, the global prevalence of CKD was 9.1%, equating to approximately 700 million cases. Reduced glomerular filtration rate (GFR), compared to other risk factors, is an independent risk factor for cardiovascular disease and overall mortality. In 2017, CKD was the 12th leading cause of death globally, marking an increase from 17th place in 1990 [3]. ESKD necessitates treatment with renal-replacement therapies (RRT), with dialysis being the most prevalent approach [2]. Other RRT modalities include conservative care [3] and kidney transplantation [4]. Kidney transplantation is considered the ideal treatment for ESKD patients, leading to a clear reduction in mortality regardless of co-morbidities and age. Therefore, the gold standard of care should prioritize achieving kidney transplantation and minimizing the need for dialysis. However, since there is currently no means to achieve immunological tolerance after kidney transplantation, lifelong immunosuppressive therapy becomes mandatory. The cornerstone of immunosuppression is based on calcineurin inhibitors, particularly cyclosporine A (CsA) and tacrolimus. Presently, CsA is rarely given to de novo kidney-transplant recipients due to its major side effects of nephrotoxicity [5], although tacrolimus is also, albeit to a lesser extent, nephrotoxic [6]. Maintenance immunosuppression in kidney transplantation, for the majority of patients, relies on tacrolimus as the primary agent in combination with mycophenolate, with or without corticosteroids [7]. A tacrolimus trough target of 5–8 ng/mL appears optimal for anti-rejection prophylaxis. However, the long-term side effects of tacrolimus and nephrotoxicity support the continual evaluation of non-calcineurin inhibitor-based regimens, such as belatacept [8]. In some patients at risk for or already experiencing post-transplant de novo cancers, mycophenolate can be replaced by mTOR inhibitors, such as sirolimus or everolimus [9].

Due to the narrow therapeutic windows of immunosuppressive drugs, many side effects can occur, some attributed to a single agent and others likely to a combination of drugs. The most prevalent side effects include calcineurin inhibitor-related nephrotoxicity, followed by hypertension and new-onset diabetes (NODAT)/post-transplant diabetes mellitus. Some of these side effects might be reversible at certain points.

In this review, we will not address all potential side effects of immunosuppressive drugs but will focus on those critical to a patient’s management and quality of life. Specifically, we will focus on nephrotoxicity, new-onset diabetes after transplantation (NODAT)/post-transplant diabetes mellitus (PTDM), leukopenia, anemia, dyslipidemia, mouth ulcers, hypertension, and viral reactivations.

### 1.1. Nephrotoxicity

Calcineurin inhibitor-related nephrotoxicity can either manifest shortly after transplantation, potentially being reversible, or occur and persist after 3–6 months post-transplantation, resulting in irreversibility. A longitudinal clinical study by Nankivell et al. [10] focused on tacrolimus nephrotoxicity, involving 119 kidney–pancreas-transplant recipients. Sequential kidney biopsies over the initial 10 years post-transplantation revealed that tacrolimus-related lesions, [11] such as striped cortical fibrosis or new-onset arteriolar hyalinosis associated with tubular microcalcification, developed in 76.4% of the patients after 1 year, 93.5% after 5 years, and 96.8% after 10 years. The authors suggested that early immune-mediated lesions followed by chronic calcineurin inhibitor nephrotoxicity contributed to kidney function/survival deterioration beyond 1 year post-transplantation.

In a subsequent study in 2016 [12], the same authors expanded their investigation to a larger cohort of 200 kidney–pancreas-transplant recipients, excluding those with pre-existing arteriolar hyalinosis. Patients receiving cyclosporine or tacrolimus were included to enable comparisons between the two calcineurin inhibitors. Despite tacrolimus inducing less frequent calcineurin inhibitor nephrotoxicity overall, arteriolar hyalinosis remained significantly associated with tacrolimus exposure, with a mean tacrolimus trough concentration of 9.4 µg/L.

Naesens et al. [13] provided an overview of CNI nephrotoxicity, elucidating the intrinsic mechanistic effects of tacrolimus on all four sectors of the kidney glomerulus, tubules, interstitium, and vessels. They suggested that local exposure to cyclosporine or tacrolimus might be more crucial than systemic exposure. Moreover, various local susceptibility factors for calcineurin inhibitor nephrotoxicity were identified, such as variability in P-glycoprotein and CYP3A4/5 expression, or activity, older kidney age, salt depletion, the use of nonsteroidal anti-inflammatory drugs, and genetic polymorphisms in genes, like TGF-beta and angiotensin-converting enzyme (ACE).

CNI-related nephrotoxicity can be either acute (and therefore reversible) or chronic, lasting for more than 3 months and potentially leading to irreversible kidney injury with eventual ESKD. Regarding tacrolimus therapy, given its very narrow therapeutic window, early post-transplant tacrolimus-related nephrotoxicity can be partially avoided by using induction therapy with either basiliximab or antithymocyte globulins to achieve lower tacrolimus trough levels, maintaining the prevention of acute rejection [14,15]. Alternatively, algorithms predicting desirable trough levels [16,17] or minimizing tacrolimus exposure [18] by replacing mycophenolate with everolimus, as seen in the TRANSFORM study [19], offer additional options. Finally, in cases of chronic tacrolimus-related nephrotoxicity, as identified in a kidney-allograft biopsy using the Banf classification [20]), tacrolimus could be easily replaced with belatacept. This conversion was deemed safe in a recent phase 3 trial [21].

### 1.2. Post-Transplant Diabetes Mellitus (PTDM)

Post-transplant diabetes mellitus (PTDM) or new-onset diabetes (NODAT) does not affect all kidney-transplant recipients. However, it is prevalent in the first weeks/months after kidney transplantation and may improve in some patients when steroids are tapered. The risk factors for PTDM are classified as modifiable and non-modifiable [22,23]. Non-modifiable factors include age, i.e., age > 40 years, with an increased likelihood that PTDM beta-cell function diminishes, leading to insulin resistance. Patients with numerous predisposing single-nucleotide polymorphisms (SNP) are more prone to develop PTDM [24]. Polymorphisms in the hepatocyte nuclear factor-4-alpha (HNF-4A) and insulin receptor substrate-1 genes are linked to PTDM development in Hispanic renal-allograft recipients [25]. The human leukocyte antigen (HLA) genotype’s role in PTDM remains unknown [26]. Additionally, a family history of diabetes mellitus is identified as a risk factor for PTDM. Recently, a meta-analysis of patients with autosomal dominant polycystic kidney disease suggested a higher risk of PTDM in those with the disease [27]. A comparative case–control study indicates that people of South Asian ancestry have a higher chance of PTDM than white people [28].

Modifiable factors include significant pre-transplantation risk factors such as body mass index (BMI) > 30, metabolic syndrome, chronic hepatitis C virus infection, prediabetes, and occult diabetes. Post-transplantation risk factors include immunosuppressant drugs like corticosteroids and tacrolimus and, to a lesser extent, cyclosporine. Conversely, belatacept-based therapy is associated with significantly less NODAT [29]. Additionally, cytomegalovirus infection [30] and hypomagnesemia [31] are also risk factors for PTDM development.

Amongst all the maintenance immunosuppressive drugs, steroids and tacrolimus are major contributors to PTDM occurrence. A systematic literature review shows that steroid-sparing and withdrawal strategies can reduce the need for antihypertensive drugs, serum cholesterol drugs, and antihyperlipidemic drugs and the occurrence of NODAT requiring treatment, as well as cataracts [32]. However, a randomized prospective controlled trial suggests that early steroid withdrawal (POD 7) has a limited impact in reducing NODAT compared to low-dose prednisone (5 mg/day), i.e., NODAT developed in 36.3% of patients on chronic corticosteroid therapy and in 35.9% of those with early corticosteroid withdrawal [33].

In a prospective non-randomized clinical trial, a late switch from tacrolimus-based to belatacept-based immunosuppression was shown to be a valuable therapeutic option for diabetic kidney recipients, substantially improving glycemic parameters. In diabetic kidney-transplant recipients, HbA1c decreased from 7.2 ± 1 to 6.5 ± 1% (*p* = 0.001). Moreover, HbA1c significantly decreased regardless of whether diabetes was controlled at inclusion or not (HA1c ≤ 7% or >7%) [34]. Therefore, in patients developing PTDM, conversion from tacrolimus to belatacept is a viable option.

PTDM treatment involves diet, oral antidiabetic agents, and insulin therapy where required (e.g., very high fasting blood-sugar levels) [35]. Novel agents, including sodium glucose co-transporter 2 inhibitors (SGLT2i), glucagon-like peptide-1 receptor agonists (GLP1RA), and dipeptidyl peptidase IV inhibitors (DPP4i), show great promise for type 2 diabetes management in the non-transplant population. However, current experience with novel antihyperglycemic agents is primarily limited to single-center retrospective studies and case series [36].

### 1.3. Haematological Disorders: Leukopenia and Anemia

Leukopenia is most likely to occur within the first months post-transplantation, whereas anemia might occur at any time post-transplantation, particularly when kidney allograft function is poor.

Leukopenia. The word leucopenia is often used interchangeably with neutropenia. Various definitions for leucopenia and neutropenia exist; however, leucopenia is graded based on the Common Terminology Criteria for Adverse Events (CTCAE) [37]. CTCAE categorizes leucopenia into four levels: grade 1 (lower range of normal limits to 3000 cells/mm^3^), grade 2 (2000–3000 WBC/mm^3^), grade 3 (1000–2000 WBC/mm^3^), and grade 4 (less than 1000 WBC/mm^3^). Most laboratories consider 4000 cells/mm^3^ as the lower limit of normal, and any level below this is considered leucopenia. Transplant physicians use neutropenia to classify granulocytopenia based on its severity, utilizing the absolute neutrophil count (ANC) for its assessment. ANC is calculated as follows: ANC = white blood cells (microliter) × percent (polymorphonuclear cells + bands)/100. An ANC < 1500/microliter or <1.5 × 109/L is defined as neutropenia categorized as mild, moderate, or severe. Mild neutropenia has an ANC in the range of 1000 to 1500/microliter or 1 to 1.5 × 109/L. Moderate neutropenia is defined as 500 to 999/microliter or 0.5 to 0.99 × 109/L. Severe neutropenia refers to ANC < 500/microliter or <0.5 × 109/L [38]. Neutrophils and lymphocytes play vital roles against infections, and leukopenic kidney-transplant recipients are prone to opportunistic infections. An ANC of less than 1000 cells per L increases susceptibility to infections. The frequency and severity of infections are increased with decreasing neutrophil counts and prolonged duration of neutropenia. *Escherichia coli* infections are more common in neutropenic kidney-transplant recipients [39]. Induction immunosuppression, such as lymphocyte-depleting polyclonal antibodies like antithymocyte globulins (ATG), may cause transient leucopenia/neutropenia. ATG is not specific for T-cells and contains antibodies directed against different blood cell types (T-cells > B-cells; NK cells > monocytes; neutrophils > platelets > erythrocytes). As a result of the presence of cross-reacting antibodies against non-lymphoid cells, hemolytic anemia, thrombosis, thrombocytopenia, and neutropenia can occur. At high doses of ATG, nonspecific binding to neutrophils and platelets can lead to undesirable effects, such as transient neutropenia and thrombocytopenia [40]. The incidence of leucopenia varies among kidney-transplant recipients due to inconsistencies in duration and dosing regimens. Incidences of leucopenia range from 10% to 50%, according to various authors (reviewed by Khalil et al. [41]). When treating ATG-induced cytopenia, consider the effects of other immunosuppressive medications, such as mycophenolate mofetil (MMF) or mycophenolic acid (MPA), substances given to almost all de novo kidney-transplant recipients. Additionally, many patients will receive valganciclovir as anti-cytomegalovirus prophylaxis and sulfamethoxazole-trimethoprim as anti-Pneumocystis jirovecii prophylaxis simultaneously [42].

A recent systematic literature review identified 73 studies reporting on the epidemiology of post-kidney-transplant leukopenia/neutropenia [43]. The pooled incidence of neutropenia (absolute neutrophil counts < 1000/mm^3^) ranged from 13% to 48% within 1 year post-transplantation, and counts <500/mm^3^ ranged from 15% to 20%. Leukopenia (white blood cell counts < 3500/mm^3^) ranged from 19% to 83%. Only 11 studies reported independent risk factors associated with post-kidney-transplant leukopenia. Donor (+)/recipient (−) cytomegalovirus serostatus, mycophenolic acid, and tacrolimus use were consistent risk factors across studies. Fourteen studies reported leukopenia/neutropenia-associated clinical outcomes. There was a trend towards a positive association between neutropenia and acute rejection/opportunistic infections. Mixed findings were noted on the association between leukopenia/neutropenia and graft failure or mortality. Dosage modifications of valganciclovir, mycophenolic acid, sulfamethoxazole-trimethoprim, and anti-thymoglobulin globulins and the need for granulocyte colony-stimulating factor (G-CSF) were common with leukopenia/neutropenia.

Mycophenolate is a cornerstone of maintenance immunosuppression after kidney transplantation; in most cases, it is given in addition to tacrolimus. Mycophenolate contributes to leukopenia, especially when it is associated with valganciclovir [44,45]. When a mycophenolate-treated patient develops neutropenia, the abbreviated area under the curve (AUC) can be ascertained to rule out overexposure [46]. Management of severe leukopenia relies on G-CSF to achieve a quick recovery of WBC count when leukocytes are needed, in addition to changes in immunosuppression and prophylaxis medications [47].

Anemia. Post-transplantation anemia is a common issue following kidney transplantation [48]. In 2003, a European survey (TRESAM study) that included 4263 kidney-transplant recipients revealed that 38.6% of recipients experienced anemia. Of the 8.5% of patients considered severely anemic, only 17.8% received treatment with epoetin. A strong association between hemoglobin and graft function was observed; among the 904 patients with serum creatinine > 2 mg/dL, 60.1% were anemic compared to 29.0% with serum creatinine ≤ 2 mg/dL (*p* < 0.01). Anemia was also more likely with therapy involving angiotensin-converting enzyme (ACE) inhibitors, angiotensin II receptor antagonists, mycophenolate mofetil, or azathioprine [49]. A recent meta-analysis indicated that post-transplantation anemia was linked to higher overall mortality (pooled risk ratio = 1.72 [1.39, 2.13], I2 = 56%), graft loss (pooled risk ratio = 2.28 [1.77, 2.93], I2 = 94%), cardiovascular death (pooled risk ratio = 2.06 [1.35, 3.16], I2 = 0%), and cardiovascular events (pooled risk ratio = 1.33 [1.10, 1.61], I2 = 0%). Early anemia (≤6 months) carried a higher risk of overall mortality and graft loss compared to late anemia (>6 months), with a pooled risk ratio of 2.63 (95% CI 1.79–3.86; I2 = 0%) and 2.96 (95% CI 2.29–3.82; I2 = 0%), respectively [50]. An open-label French multicenter randomized controlled trial investigated the effect of epoetin-β on normalizing hemoglobin values (13.0–15.0 g/dL, n = 63) versus partial correction of anemia (10.5–11.5 g/dL, n = 62) in transplant recipients with hemoglobin < 11.5 g/dL and estimated creatinine clearance (eCrCl) < 50 mL/min per 1.73 m^2^. After 2 years, mean hemoglobin was 12.9 and 11.3 g/dL in the normalization and partial-correction groups, respectively (*p* < 0.001). From baseline to year 2, eCrCl decreased by a mean of 2.4 mL/min per 1.73 m^2^ in the normalization group compared to 5.9 mL/min per 1.73 m^2^ in the partial-correction group (*p* = 0.03). Furthermore, fewer patients in the normalization group progressed to end-stage kidney disease (3 vs. 13, *p* < 0.01). The cumulative death-censored graft survival was 95% and 80% in the normalization and partial-correction groups, respectively (*p* < 0.01). Finally, complete correction was associated with a significant improvement in quality of life at 6 and 12 months [51]. Iron deficiency is highly prevalent in kidney-transplant recipients and independently associated with a higher mortality risk in this population.

Various factors contribute to iron deficiency in kidney-transplant recipients, including inflammation, medication, and an increased need for iron after transplantation [52]. Recent evidence suggests that routine parenteral iron treatment after kidney transplantation is linked to a lower prevalence of early- and late-onset anemia and a reduced requirement for erythropoietin-stimulating rescue agents or blood transfusions [53].

Based on these data, it is imperative to recognize and to treat post-transplant anemia.

### 1.4. Dyslipidemia

Dyslipidemia is prevalent within the first months post-transplantation and can improve significantly when daily steroid dosing is reduced to lower levels. Immunosuppressive medications are associated with dyslipidemia, and kidney-transplant recipients face numerous factors contributing to cardiovascular risks. The pathogenesis of cardiovascular disease after kidney transplantation is multifactorial, involving non-modifiable risk factors (age, gender, genetic predisposition, and ethnicity) as well as traditional and non-traditional modifiable risk factors. Traditional factors, such as diabetes, hypertension, and dyslipidemia may be present before transplantation and may worsen afterward. Immunosuppressants and impaired graft function may strongly influence the exacerbation of these co-morbidities [54]. Calcineurin inhibitors, integral to post-transplant immunosuppression, have known associations with dyslipidemia. Cyclosporine A (CsA) usage is recognized to be dose-dependent, leading to increased total cholesterol and low-density lipoprotein (LDL) cholesterol, decreased high-density lipoprotein (HDL) cholesterol, and elevated serum triglycerides [55]. Tacrolimus is associated with a similar but milder dyslipidemia profile compared to CsA [56]. CsA is linked to an increase in oxidized LDL, posing a higher risk of atherosclerosis, whereas data regarding tacrolimus’ effect on LDL oxidation are mixed [57,58].

Most kidney-transplant recipients receive calcineurin inhibitors along with mycophenolate mofetil. However, in some cases, mycophenolate mofetil can be replaced by inhibitors of the mechanistic target of rapamycin (mTOR), such as sirolimus or everolimus. These mTOR inhibitors exhibit unique anti-atherosclerotic effects, including depletion of plaque macrophages, induction of autophagy, and activation of cholesterol efflux. Nonetheless, a common side effect of their use is dyslipidemia, a well-known risk factor for atherosclerosis. mTOR inhibitors prevent lipid storage, increase low-density lipoprotein cholesterol levels, and activate lipolysis. Although the net effect of mTOR inhibition seems favorable, the use of cholesterol-lowering drugs to manage dyslipidemia remains the most recommended strategy [59]. Despite the increase in serum lipids, mTOR inhibitors are associated with an overall lower risk of atherosclerosis [60]. Both mycophenolate mofetil and azathioprine appear to have a neutral effect on lipids, with no significant changes observed in lipid profiles in clinical studies [61]. Currently, statins are the pharmacologic intervention of first choice if lifestyle changes fail adequately to lower LDL-C levels in the setting of normal or moderately elevated triglycerides. Statins, extensively studied in various patient populations, have proven efficacy in treating dyslipidemia and reducing cardiovascular mortality [62]. However, side effects (e.g., myopathy) may occur. In such cases, ezetimibe (which does not affect kidney function) alone or with statins can be given for severe cases, as suggested by recent guidelines [63]. Hepatic transaminase elevations may occur in 1 to 2% of statin-treated patients and are dose-related. Myalgia, myopathy, and rhabdomyolysis occur infrequently and are more common in kidney-transplant recipients and patients with chronic kidney disease. This effect appears to be dose-related and may be precipitated by administration with agents that inhibit cytochrome P-450 isoenzymes [64].

### 1.5. Mouth Ulcers

Mouth ulcers are most often observed after the onset of mTOR inhibitor treatment and typically improve in most cases afterwards. While not commonly observed after kidney transplantation, when they occur, they may negatively impact the patient’s quality of life. Sarmento et al. recently published a prospective observational cohort study involving 80 adult kidney-transplant recipients [65]. Patients were evaluated for mouth lesions at three different times: 24 h before transplantation (first time point), 15–20 days after (second time point), and 45–60 days after transplantation (third time point). Sarmento et al. found that 3.7% (3/80), 23.7% (18/76), and 25.7% (19/74), respectively, of participants had oral soft-tissue lesions. Ulcers and candidiasis were the most frequent oral lesions and were associated with the use of everolimus (*p* = 0.005) and azathioprine (*p* = 0.034), respectively. It is well-known that both sirolimus [66,67] and everolimus, i.e., mTOR inhibitors, can cause mouth ulcers. The initial evidence came from a randomized multicenter trial involving 33 steroid-free kidney-transplant recipients receiving a steroid-free maintenance treatment of tacrolimus and mycophenolate mofetil. At 1 year post-transplantation, they were randomized either to continue tacrolimus and mycophenolate mofetil (control group, n = 18) or to switch from tacrolimus to sirolimus (study group, n = 15). The study was prematurely stopped due to a cluster of nine patients (50%) in the study group experiencing painful oral ulcerations. Oral ulcerations did not occur in the control group [66]. In another case study, sirolimus-related mouth ulcers were observed in 21.6% of patients. Histological examination revealed non-specific ulcerations associated with a polymorphous inflammatory infiltrate. Topical treatment with clobetasol reduced pain and shortened healing times between two- and three-fold [67].

In the setting of liver transplantation, a single-center, randomized, controlled trial, was conducted, including 30 maintenance patients randomized to remain on calcineurin (CNI)-based immunosuppression or be switched to sirolimus-based immunosuppression. They observed that one-third of patients converted to sirolimus experienced aphthous-type mouth ulcers that resolved over the course of the first 2 weeks with dose adjustment to the lower end of the target range, although three patients developed coincidental herpes simplex [68]. Everolimus is also associated with mouth ulcers [69,70] and perianal ulcers [70,71]. It is often dose-related, especially in those on steroid-free immunosuppression and after conversion from CNIs.

Management of mTOR inhibitor-related mouth ulcers relies on minimizing (where possible) trough levels, increasing (or adding) systemic steroids, and applying topical clobetasol [67]. In the setting of oral chronic graft-versus-host disease (GVHD), a randomized, double-blind clinical trial compared topical clobetasol and dexamethasone. It was found that clobetasol was significantly more effective than dexamethasone at ameliorating symptoms and clinical aspects of oral lesions in chronic GVHD [72].

### 1.6. Hypertension

Hypertension is highly prevalent after kidney transplantation and is observed from the first weeks post-transplantation. The 2021 Kidney Disease Improving Global Outcomes BP guidelines defined hypertension as office blood pressure (BP) of ≥ 130/80 mmHg and ambulatory BP monitoring (ABPM) as ≥125/75 mmHg, in agreement with the 2017 ACC/AHA guidelines [73]. In a recent cohort study of 260 kidney-transplant recipients followed-up for 3.9 years, the agreement between 785 paired office and 24 h ABPM measurements was assessed, revealing significant discordance in 37% of all visits (κ-statistic = 0.25, indicating poor agreement) [74]. The prevalence of post-kidney-transplant hypertension ranges between 55% and more than 95% [75,76].

Among traditional and non-traditional risk factors, post-transplant hypertension remains a major contributor to post-transplantation cardiovascular morbidity and mortality, as well as one of the most common causes of chronic graft dysfunction and kidney-transplant failure [75]. Cardiovascular pathology is responsible for approximately 40% of deaths among kidney-transplant recipients [77]. Therefore, recognizing and treating post-kidney-transplant hypertension is crucial. There are various non-modifiable and modifiable factors, including those associated with immunosuppression (cyclosporine, tacrolimus, glucocorticoids), the allograft (delayed graft function, chronic allograft nephropathy, de novo and recurrent glomerular disease, acute rejection), the recipient (underlying kidney disease, essential hypertension, native kidney presence, excessive weight gain, secondary hyperparathyroidism), donor factors, and surgery (e.g., renal-allograft artery stenosis). Once post-kidney-transplant hypertension is identified and a treatable cause is ruled out (or managed where present), the KDIGO guidelines recommend the use of a calcium-channel blocker or an angiotensin-receptor blocker as the first-line antihypertensive agent (1C) [73]. The nocturnal use of calcium-channel blockers has shown good performance in reversing the altered sleep patterns; they are especially indicated in the first few months after renal transplantation when the risk of ureteric stricture is maximal. Additionally, they are preferable because the risk of acute kidney injury is greater when angiotensin II AT1 receptor blockers are used; therefore, the latter are only to be used when renal function is stable [78].

### 1.7. Viral Reactivation

In most cases, viral reactivations occur within the first months after kidney transplantation. Induction and maintenance immunosuppression can lead to impaired immune responses, including antiviral responses, favoring opportunistic infections, such as cytomegalovirus (CMV) and BK virus (BKV) infections [79,80]. These infections can impair graft function and contribute to transplant rejection.

Cytomegalovirus is frequently reactivated after kidney transplantation. Two main approaches to prevent CMV reactivation are universal prophylaxis with valganciclovir and preemptive therapy, where CMV DNAemia is regularly monitored and valganciclovir therapy is initiated upon a positive result. A recent literature review revealed that despite preventative approaches, approximately one-fourth of kidney-transplant recipients developed CMV infection. Age and D+/R− CMV serostatus were consistent risk factors for CMV infection/disease, which, in turn, was associated with increased mortality and graft loss [81].

Recently, Reischig et al. reported on an open-label, single-center, randomized clinical trial of valganciclovir prophylaxis versus preemptive therapy in 140 de novo kidney-transplant recipients (CMV-seronegative recipients excluded) with transplants from seronegative donors. Patients were randomized 1:1 to receive preemptive therapy (valganciclovir, 900 mg twice daily) or valganciclovir prophylaxis (900 mg daily for 3 or 6 months for CMV-seronegative recipients with a CMV-seropositive donor). Preemptive therapy was initiated after the detection of CMV DNA in whole blood (≥1000 IU/mL) and stopped after two consecutive negative tests (weekly CMV PCR tests for 4 months). The primary outcome was the incidence of biopsy-confirmed acute rejection at 12 months. The study observed a lower incidence of acute rejection with valganciclovir prophylaxis compared to preemptive therapy (13%, 9/70 vs. 23%, 16/70), and subclinical rejection at 3 months was lower in the prophylaxis group (13% vs. 29%, *p* = 0.027). Both regimens prevented CMV disease (4% of patients in both groups). Preemptive therapy, compared with universal prophylaxis, resulted in significantly higher rates of CMV DNAemia (44% vs. 75%, *p* < 0.001) and a higher proportion of patients experiencing episodes with a higher viral load (≥2000 IU/mL) [82].

When a kidney-transplant recipient experiences a first CMV infection episode, the question arises as to whether maintenance immunosuppression should be modified. Previous studies have demonstrated the anti-CMV properties of mTOR inhibitors such as sirolimus and everolimus [83,84]. In a recent single-center prospective randomized trial conducted by Viana et al. with a 12-month follow-up, 72 kidney-transplant recipients who had undergone treatment for a first episode of CMV infection/disease either were maintained on the same treatment (antiproliferative agent plus CNIs) or had the antiproliferative replaced with sirolimus. The study found that CMV recurrence occurred in 43% of patients in the control group, whereas the sirolimus group had no recurrence (*p* ≤ 0.0001) [85]. In de novo kidney-transplant recipients, avoiding CMV infection/disease can be achieved to some extent by replacing the antiproliferative agent (mycophenolic acid) with everolimus [19,86].

Poliomavirus BK virus (BKV) is highly infective and causes asymptomatic infections during childhood. After the initial infection, BVK establishes a stable state of latent infection in kidney tubular cells and the uroepithelium with negligible clinical consequences. However, BKV isa significant risk factor for BKV-associated diseases, including BKV-associated nephropathy (BKVN), in kidney-transplant recipients. BKVN affects up to 10% of renal-transplant recipients and results in graft loss in up to 50% of those affected. Unfortunately, treatments for BK virus infection are restricted, and there is no efficient prophylaxis [87,88,89]. Due to the lack of efficient BKV prophylaxis, regular monitoring for BKV replication in the blood and urine is essential, especially during the first year post-kidney transplantation and whenever serum creatinine changes subtly with no identifiable cause.

In a recent prospective cohort of 540 de novo kidney-transplant recipients reported by Blasquez-Navarro et al. BKV, CMV, and Epstein-Barr virus (EBV) viral loads were analyzed using qPCR throughout eight visits during the first post-transplantation year [90]. BKV had the highest prevalence and viral loads. BKV viral loads exceeding 10,000 copies·mL^−1^ led to a significant impairment of the estimated glomerular filtration rate (eGFR). Both BKV and CMV reactivations were significantly associated (*p* = 0.005). Additionally, combined reactivation was associated with a significant reduction in eGFR at 1 year post-transplantation of 11.7 mL·min^−1^·1.73 m^−2^ (*p* = 0.02) or relatively low thresholds (BKV > 1000 and CMV > 4000 copies·mL^−1^) [90].

It has been demonstrated that the use of the mTOR inhibitor everolimus, replacing mycophenolate acid, and in association with low doses of calcineurin inhibitors in de novo kidney-transplant recipients, resulted in significantly less BKV replication [19]. In the TRANSFORM study, BK virus infection (viruria or viremia) occurred in 4.3% and 8.0% of everolimus- and mycophenolic acid-treated patients, respectively (RR, 0.54; 95% CI, 0.38 to 0.77), with histologic evidence of organ involvement in 1.2% (12/1014) and 2.1% (21/1012), respectively.

On the other hand, when BKV replication is already present, several options are available. The first option is to minimize stepwise immunosuppression by reducing or eliminating mycophenolic acid [91,92] or converting mycophenolic acid to everolimus with calcineurin inhibitor minimization [92,93]. Patients with high titers of BKV-neutralizing antibodies (NAbs) are protected against BKV replication, and intravenous immunoglobulin (IVIg) infusion can increase NAb titers. Therefore, based on NAb titers on the day of kidney transplantation, patients with low NAb titers have a greater risk of BKV reactivation. Benotmane et al. demonstrated that 12 months after transplantation, the incidence of BKV viremia in the high-risk group treated with prophylactic IVIg (6.8%) was similar to that observed in the low-risk group (10.1%) that received no IVIG and was markedly lower than that of the untreated high-risk group (36.6%; *p* < 0.001). Additionally, Benotmane et al. found similar results regarding BKVAN [94]. Anyaegbu et al. showed that IVIg was an effective treatment for persistent BKV infection after a reduction in immunosuppression and for BKVN [95]. Another option for overt BKV-associated nephropathy is calcineurin inhibitor-free immunosuppression based on either everolimus [96] or leflunomide [97,98].

### 1.8. Fertility

mTOR serves as a central key regulator of cell growth, proliferation, and nutritional status, influencing growth factor signaling pathways in mammalian cells. In testes and testicular cells, numerous histological and molecular events are mediated by mTOR [99].

Despite their common side effects, sirolimus and everolimus may have negative impacts on sex hormone levels and spermatogenesis in patients after solid organ transplantation. Studies have demonstrated that sirolimus can lead to a significant decrease in testosterone, along with a significant increase in follicle stimulating hormone (FSH) and luteinizing hormone (LH). The duration of sirolimus treatment and sirolimus trough levels have shown a positive correlation with impaired sex hormone binding globulin and LH and FSH levels and a negative correlation with the free androgen index [100]. Withdrawal of sirolimus as part of maintenance immunosuppression has been reported to improve sperm quality and sex hormone parameters [101].

In female patients, immunosuppression treatment with mTOR inhibitors has been associated with the development of ovarian cysts as a common adverse event. While these cysts are typically benign, they necessitate pelvic ultrasound follow-up, and in some cases, hospital admission and surgery may be required [102].

### 1.9. Wound Complications

mTOR inhibitors might interfere with wound-healing processes. A meta-analysis revealed that the use of mTOR inhibitors post-solid organ transplantation was correlated with an increased occurrence of wound complications and lymphoceles after kidney transplantation, as well as a heightened frequency of wound complications subsequent to heart transplantation, especially in immunosuppressive regimens incorporating mTOR inhibitors from the time of transplantation [103]. However, these studies were conducted during a period when exceptionally elevated trough levels of sirolimus or everolimus were targeted. Presently, the standard practice in the use of mTOR inhibitors involves aiming for lower everolimus trough levels. This adjustment did not lead to a higher incidence of wound-healing complications compared to mycophenolic acid in patients undergoing CNI-based immunosuppression after kidney transplantation [103].

### 1.10. Urinary Tract Infection (UTI)

Asymptomatic bacteriuria (ASB) is prevalent in kidney-transplant recipients and is hypothesized to heighten the risk of subsequent urinary tract infections. Whether antibiotic treatment of ASB in kidney-transplant recipients is beneficial has not been elucidated. Recently, a systematic review and meta-analysis of all randomized controlled trials (RCTs) and quasi-RCTs was conducted. It assessed the merits of managing asymptomatic bacteriuria in kidney-transplant recipients. The primary outcomes were rates of symptomatic urinary tract infections (UTIs) and antimicrobial resistance [104]. There were five studies encompassing 566 patients. They found that the practice of screening and treating kidney-transplant patients for asymptomatic bacteriuria does not curtail the incidence of future symptomatic UTIs, increase antimicrobial resistance, or affect graft outcomes. In a single-center retrospective study of 207 consecutive kidney transplant recipients, Strohaeker et al. showed that urinary tract infections appear to be linked to worse graft functions, thus leading to the recommendation of prevention and treatment of them [105].

### 1.11. De Novo Post-Transplant Malignancies

It is well-known that after kidney transplantation, the risk of developing de novo cancers is increased, particularly for those that are virus-induced, e.g., skin cancers, cervical cancer, post-transplant lymphomas, Kaposi’s sarcomas, etc. [106]. A recent study from Italy analyzed 25-year variations in cancer incidence among 11,418 Italian recipients of kidney transplants. The cancer incidence was examined over three periods (1997–2004; 2005–2012; and 2013–2021). It was found that there was a decline in standardized incidence ratios (SIR) specifically for non-Hodgkin’s lymphoma and Kaposi’s sarcoma, though only the Kaposi’s sarcoma trend retained statistical significance after adjustment. The authors concluded that, apart from Kaposi’s sarcoma, no changes in the incidence of other cancers over time were observed among kidney-transplant recipients [107].

Table 1 summarizes the principal side effects, their mechanisms of action, and the therapeutic options.

## 2. Conclusions

After kidney transplantation, lifelong immunosuppression is imperative to achieve favorable long-term results, as there is no immune tolerance established. Maintenance immunosuppression relies on a combination of drugs with narrow therapeutic windows, often resulting is adverse drug events. These events may contribute to allograft failure (e.g., nephrotoxicity, hypertension) or increase the risk of cardiovascular disorders (e.g., dyslipidemia, hypertension, post-transplant diabetes mellitus), as well as drug discontinuation, as seen in mTOR inhibitor-induced mouth ulcers. Certain side effects, such as hypertension, nephrotoxicity, or PTDM, can be significantly improved either by substituting CNI with belatacept or by minimizing CNI through the replacement of mycophenolic acid with low doses of everolimus.

## Figures and Tables

**Table 1:** Most frequent adverse drug events after kidney transplantation.

Side Effects	Mechanisms	Therapeutical Options
Nephrotoxicity	Vasoconstriction induced by CNI	CNI exposure decrease Conversion from CNI- to belatacept-based therapy ***Or*** CNI minimization/MPA withdrawal/everolimus introduction
Hypertension	CNI-induced vasoconstriction	Calcium channel blockers CNI dose reduction
PTDM	Induced by CNI (Tac >>> CSA)	Tac lowering Replacement of Tac by belatacept
Leukopenia	MPA-indcued, mTOR-I-induced, or valganciclovir-induced	Transient reduction in MPA or everolimus exposures Growth factors (GCSF)
Anemia	MPA- or mTRO-I-induced Associated with CKD	Recombinant erythropoïetin
Dyslipidemia	CNIs (CSA >> Tac)/steroids/mTOR-is	Statins
Mouth ulcers	mTOR-Is	Decrease mTOR-I exposure Topical clobetasol
Viral reactivation	Immunosuppression	Decrease immunosuppression Specific therapies
Fertility	mTOR-Is inhibit spermatogenesis	Withdraw mTOR-Is where possible
Wound complications	mTOR-Is	mTOR-Is minimization

Abbreviations: CNI, calcineurin inhibitor; mTOR-Is, mTOR inhibitors; MPA, mycophenolic acid; PTDM, post-transplant diabetes mellitus; Tac, tacrolimus; CSA, cyclosporine A.

## Data Availability

Not applicable.
